# Hyperthyroxinemia with a non-suppressed TSH: how to confidently reach a diagnosis in this clinical conundrum

**DOI:** 10.1007/s42000-020-00180-3

**Published:** 2020-03-03

**Authors:** J. G. Timmons, B. Mukhopadhyay

**Affiliations:** 1 Department of Diabetes and Endocrinology, University Hospital Hairmyres, East Kilbride, Glasgow, UK; 2 British Heart Foundation Cardiovascular Research Centre, Institute of Cardiovascular and Medical Sciences, University of Glasgow, 126 University Place, Glasgow, G12 8TA UK

**Keywords:** Discordant thyroid function tests, Resistance to thyroid hormone (RTH), TSH-secreting pituitary adenoma, TSHoma, Central hyperthyroidism

## Abstract

Disorders of thyroid function are among the commonest referrals to endocrinology. While interpretation of thyroid function testing is usually straightforward, accurate interpretation becomes significantly more challenging when the parameters do not behave as would be expected in normal negative feedback. In such cases, uncertainty regarding further investigation and management arises. An important abnormal pattern encountered in clinical practice is that of high normal or raised free thyroxine (fT4) with inappropriately non-suppressed or elevated thyroid-stimulating hormone (TSH). In this short review using two clinical vignettes, we examine the diagnostic approach in such cases. A diagnostic algorithm is proposed to ensure that a definitive diagnosis is reached in these challenging cases.

## Learning point 1

In a patient who is clinically euthyroid with elevated fT4 and an inappropriately normal or raised TSH, the most likely cause is assay interference. Interference should firstly be excluded before considering rarer conditions such as resistance to thyroid hormone and TSHoma.

## Learning point 2

Repeat thyroid function tests should be sent to another laboratory using a different assay to unmask assay interference if this is suspected.

## Learning point 3

Focused drug history and family history of thyroid function abnormalities should be specifically sought in these patients.

## Learning point 4

When assay interference has been excluded, consideration should be given to resistance to thyroid hormone (RTH) and TSH-secreting pituitary adenoma (TSHoma). Biochemical, dynamic testing, and imaging can be used to differentiate between the two conditions. An algorithmic approach to investigation as herein presented ensures that a diagnosis is reached in this clinical conundrum.

## Vignette 1

A 41-year old female was referred to the endocrinology clinic with an elevated free T4 and a non-suppressed TSH. She had initially presented to her general practitioner with lethargy. Her GP performed routine blood tests, including thyroid function tests (TFTs), which revealed normal thyroid-stimulating hormone (TSH) 0.82 mU/l (normal range 0.3–4.0 mU/l), but raised free thyroxine (fT4) 30 pmol/l (normal range 13–23 pmol/l). Total triiodothyronine (T3) was 1.3 nmol/l (normal range 1.3–3.1 nmol/l). Other biochemistry laboratory tests including renal function, liver function, plasma glucose, and lipids were normal. On specific questioning, she reported a vague history of heat intolerance and thin hair, but there were no other specific symptoms to suggest thyrotoxicosis. There was no history of menstrual irregularity, she was not taking any regular medications, and there was no personal or family history of thyroid disease. She was clinically euthyroid and the thyroid gland was unremarkable on examination. Repeat TFTs showed a similar pattern with normal range TSH and raised fT4. Antithyroid peroxidase (anti-TPO) and TSH receptor (TRAb) antibodies were not detected.

The vague symptoms this patient presents with may or may not be attributed to thyroid disease. In the general population, the commonest cause of this pattern of TFTs is irregular compliance with prescribed levothyroxine in a patient who has established hypothyroidism. TFTs repeated at the outpatient clinic—using the same assay—produced a similar result. Given that assay interference is well documented, a further sample was sent to a different laboratory using a different TFT assay with subsequently normal results. The patient was reassured that the abnormal results were due to assay interference. There was no clinical need for drug therapy or further investigation and she was discharged from the clinic.

We now explore the common causes for spurious thyroid function test results similar to those described in *Vignette 1.*

### Assay interference

Assay interference in thyroid function testing has been estimated as occurring in up to 1% of all tests performed. Given the widespread testing of thyroid function in clinical practice, assay interference is an important consideration [1]. The issue of assay interference arises because there are several different methods used to measure TSH, total and free thyroxine (T4), and total and free triiodothyronine (T3), including radioimmunoassay, immunometric assay, and tandem mass spectrometry, but differences in sensitivity, specificity, and standardization can result in significant variability between these methods. Different manufacturers use differing methods for measurement. The gold standard free T4 assay is one that uses the equilibrium dialysis method although this is not widely available [1, 2]. The fT4 assay platforms in routine use are not always robust and can be affected by serum antibodies. The commonest cause of a falsely high TSH is assay interference from human anti-murine antibodies (HAMA) or thyroid hormone autoantibodies (THAAbs) [1, 2]. Rheumatoid factor (RF) is an antibody directed against human IgG and is often present in people with autoimmune disease—particularly rheumatoid arthritis. This can also interfere with TFT assays and should be considered if the clinical picture is suggestive [1]. Other antibodies such as anti-streptavidin and anti-ruthenium antibodies can also cause assay interference [1]. Biotin is used with increasing frequency as a health supplement and can interfere with certain TFT assays: this should be considered in the clinical history [3, 4]. Since some analytical methods are more susceptible to the effects of assay interference than others, this issue can generally be identified by remeasuring both TSH and fT4 using a different manufacturer’s assay platform [2]. In practice, this will usually involve sending samples to another lab with a different TFT assay. In practical terms, if a practitioner is unsure about the logistics of this, the local clinical biochemist would be helpful in the organizational aspects of redirecting a sample for an alternative assay measurement. If when using a different analytical method, the TFTs are normal, it suggests that assay interference is the underlying problem and the patient can be reassured [5].

### Drugs

Having considered assay interference as a spurious cause for inappropriately non-suppressed TSH with high normal or high fT4, we now consider drug causes which should be specifically sought in patients presenting with such biochemistry. Several drugs are known to cause discordant TFTs similar to those presented in *vignette 1.* As already alluded to, levothyroxine if taken irregularly mostly results in a raised TSH with normal free T4 levels; however, if erratic or inappropriate doses of thyroxine are taken, the free T4 can also be high. Aside from the overt effects of amiodarone on the thyroid gland itself, amiodarone also inhibits extrathyroidal conversion of T4 to T3 as can glucocorticoids and propranolol also. This results in a reduced T3, increase in reverse T3 (rT3), and raised T4 (although this can also be normal) with a non-suppressed TSH. Heparin displaces T4 from thyroid-binding globulin (TBG), resulting in an apparently inappropriately high free T4 on TFTs [5].

### Genetic causes

Changes affecting one of the thyroid hormone binding proteins, such as thyroid binding globulin (TBG), transthyretin, or albumin, can also affect the free T4 assay as is the case in familial dysalbuminemic hyperthyroxinemia (FDH). This is a rare genetic condition in which albumin has a preferential affinity for T4, which can lead to alterations in total and free T4 levels with non-suppressed TSH [2, 5, 6].

## Vignette 2

A 39-year old woman was referred to our clinic having attended her GP with back pain, abdominal discomfort, and bloating. She was incidentally found to have abnormal thyroid function tests. Biochemistry revealed a TSH of 1.66 mU/l (0.3–4.0 mU/l), free T4 45 pmol/l (13–23 pmol/l), and total T3 3.2 nmol/l (1.3–3.1 nmol/l). When reviewed in the clinic, she was tachycardic (heart rate 120/min regular) and was found to have a small, uniform goiter. No other abnormalities were found. TFTs were firstly repeated using a different assay and results were similar. Thyroid autoantibodies were not detected. Renal function, liver function, plasma glucose, and lipids were normal. There was no family history of thyroid disease.

**Fig 1:**
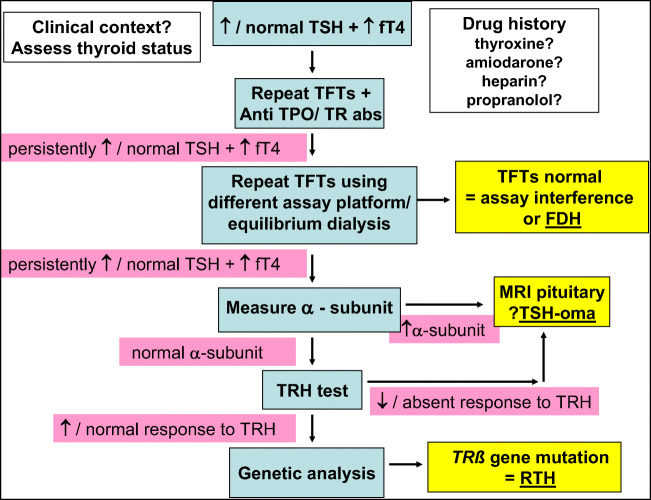
A Practical clinical algorithm for discordant TFTs

In this case, the patient has some objective features of thyrotoxicosis, notably a resting tachycardia. Since repeat TFTs using a different assay still demonstrated a discordant pattern with raised fT4 and inappropriately normal TSH, the patient was offered further endocrine investigation. (Fig. 1).

In this clinical context having excluded any obvious spurious cause for TFT derangement, two rare conditions must be considered: TSH-secreting pituitary adenoma (TSHoma) and resistance to thyroid hormone (RTH) with loss of function in the thyroid hormone receptor beta gene (TRβ). The differentiation between these conditions is often difficult.

### TSH-secreting pituitary adenoma

TSH-secreting pituitary adenoma (TSHoma) is a rare condition with a prevalence of approximately one per million [7, 8]. They are the least common form of functional pituitary adenoma accounting for only around 2% of all pituitary tumors diagnosed [7, 8]. The incidence of TSHoma has increased, likely due to improvements in biochemical and imaging modalities allowing more accurate diagnosis [9–12]. While these tumors are almost invariably benign, they are often large invasive fibrous tumors at the time of resection. A total of 90% are macroadenomas, and as such may cause local mass effect and headache. The majority (approximately two-thirds) demonstrate invasion into local structures at the time of diagnosis. While between 70 and 80% of these tumors secrete only TSH, approximately 25% are mixed and co-secrete growth hormone or prolactin [9–12]. In such cases, hypersecretion of these other anterior pituitary hormones may dominate the clinical picture. Patients generally present however with typical symptoms of thyrotoxicosis. There is often a goiter on examination. Visual field defects should be sought and formal visual field testing should be undertaken given the propensity of these tumors to be large and locally invasive at diagnosis. Hypogonadotrophic hypogonadism may also rarely be present and in females, oligomenorrhea or amenorrhea may be present [5, 8, 9].

The treatment of choice is transphenoidal resection of the pituitary adenoma following pituitary multidisciplinary team discussion. If surgery is contraindicated or declined, pituitary radiotherapy and medical treatment with somatostatin analogs can be used to suppress TSH secretion in 80% of TSHomas [7–12].

### Resistance to thyroid hormone

Resistance to thyroid hormone (RTH) is the second condition which must be considered when presented with this pattern of TFTs. RTH was first described in 1967 and is estimated to occur in around 1/40,000 to 1/50,000 live births [5]. In around 80% of cases, the mutation is inherited in an autosomal dominant fashion. The remainder of cases are due to a de novo mutation. It is primarily an inherited syndrome of reduced end-organ sensitivity to thyroid hormone which specifically pertains to defects of the thyroid hormone receptor [13]. This results in reduced intracellular action of the active thyroid hormone T3. The resistance to thyroid hormone can either be general (GRTH) or selective pituitary resistance to thyroid hormone (PRTH) [13, 14]. This can alter the clinical manifestation: if the pituitary is predominantly resistant but the peripheral tissues are not, patients may present with clinical features of thyrotoxicosis. The majority (around 85%) of patients with RTH have mutations in the thyroid hormone receptor β (TR*ß)* gene, resulting in dysfunctional beta receptors, which have also been found to diminish the activity of normal receptors, explaining the unresponsiveness of end-organ tissues to thyroid hormone [14]. Clinical manifestations are heterogeneous, but with the exception of goiter are often absent. When present, clinical signs may include coexistent paradoxical signs of thyroid underactivity and overactivity, including goiter (the most common finding), hyperactivity, learning disabilities, growth delay, and resting tachycardia. A number of people with RTH however may be relatively asymptomatic or present with mild symptoms and therefore diagnosis is frequently made in adulthood following incidental thyroid function testing or investigation of tachyarrythmia [5, 13, 14].

Treatment is generally not required for RTH although may be required symptomatically in selective pituitary resistance to thyroid hormone. Beta blockers can be used to treat thyrotoxic symptoms [5, 13, 14].

Given the heterogeneity and overlap in the clinical manifestations of both of these conditions, it is not possible to distinguish between RTH and a TSH-secreting pituitary adenoma from clinical history or examination alone. Similarly, the TSH and fT4 levels can often overlap between RTH and TSH-producing pituitary adenoma; therefore, biochemistry cannot reliably be used to differentiate between the two conditions either. TSH may be within normal range or elevated in TSHoma and in RTH. Arguably, the best biochemical investigation in the initial differentiation of TSHoma and RTH is serum glycoprotein alpha subunit (α-GSU). Alpha subunit is high in about 70% of patients with TSHoma. It is ordinarily not elevated in RTH; however, in approximately 1 in 20 patients with selective pituitary RTH, it can be elevated. Alpha subunit elevation is due to an unbalanced hypersecretion of alpha subunit in TSHoma. This is particularly true in macroadenomas which account for the majority of such tumors [5].

The TRH (thyrotropin-releasing hormone) stimulation test is useful in differentiating between TSHoma and RTH. Two hundred micrograms of TRH is given intravenously with sampling at 0, 20, and 60 min. A normal or exaggerated response to TRH is typically seen in RTH with a peak TSH of greater than fivefold baseline. This can be used to complement subsequent genetic studies if this diagnosis is suspected. Conversely, in TSHoma, an attenuated or absent TSH response is seen, but with generally less than a 1.5-fold rise in TSH from baseline in macroadenomas, although a greater response may paradoxically be seen with microadenomas [7–12].

Although not used in this case, T3 suppression testing can also be used to differentiate between TSHoma and RTH. A total of 80 to 100 μg per day is given in three divided doses over a 10-day period with beta-blockers. TSH sampling at day 0, day 5, and day 10 is undertaken. In TSHoma, TSH suppression is typically not seen despite T3 administration, while in RTH (both PRTH and GRTH) suppression of TSH typically occurs. Another mechanism for differentiating the two conditions is octreotide suppression testing. Octreotide (long-acting release) is administered at a dose of 20 mg per month over a 2-month period. In approximately 95% of people with TSH-secreting pituitary adenoma, a fall in TSH is seen with this regimen. There is no fall in TSH in resistance to thyroid hormone [7, 12].

Imaging of the pituitary gland with MRI scanning should be considered if initial biochemical findings suggest TSHoma. Imaging will typically demonstrate a macroadenoma, which accounts for 80% of TSHomas. Given the propensity for macroadenoma, formal perimetry testing should be conducted. A small proportion of these tumors are microadenomas and rarely ectopic TSH-secreting tumors have been described. The possibility of an incidental pituitary adenoma in the context of RTH may further confound diagnosis given the prevalence of this in the general population; therefore, care must be taken to ensure that a correct diagnosis is reached on the basis of biochemistry, dynamic testing, genetic analysis, and imaging [5, 7, 13, 14].

In this patient, the algorithmic approach presented (Fig. 1) was used to reach a definitive diagnosis of RTH. A TRH test was performed. This demonstrated a normal pituitary response to TRH stimulation (Table 1). Alpha subunit measurement was within the normal range at 1.9 IU/l (< 3.0 IU/l). This was consistent with a diagnosis of resistance to thyroid hormone. RTH was subsequently confirmed by genetic testing (fluorescent sequencing analysis of exon 9), which showed thyroid hormone receptor beta (TR*ß*) mutation. This causes predominant pituitary resistance to thyroid hormone as opposed to generalized resistance [13, 14].

**Table 1:** Vignette 2 TRH test results

Time (min)	TSH (mU/l)	fT4 (pmol/l)
0	1.47	47
20	14.17	44
60	8.81	51

TRH (thyrotropin-releasing hormone) stimulation test for the patient in vignette 2. Two hundred micrograms of TRH is administered intravenously. TSH levels are then measured at 0, 20, and 60 min. The greater than fivefold increase in TSH is highly suggestive of resistance to thyroid hormone. In TSHoma, an attenuated or absent response is seen (generally no greater than 1.5-fold increase in TSH)

The diagnosis and consequences of thyroid hormone resistance was explained to the patient, and genetic counseling offered. Genetic testing for her siblings was refused. Given the autosomal dominant nature of this condition, the presence of similar biochemical phenotype in first degree relatives is highly suggestive of RTH and family screening should be offered.

## Conclusion

In both cases, fT4 is elevated and TSH is inappropriately non-suppressed. Since assays can have variable performance, a logical approach needs to be taken when TSH levels are non-suppressed in the presence of elevated free T4 levels. We propose an algorithm (Fig. 1) for the management of such discordant thyroid function tests accompanied by two practical clinical examples to illustrate its use in practice. When there is raised fT4 with an inappropriately normal or raised TSH, firstly exclude assay interference before considering further investigation as per our proposed algorithm.

## Abbreviations

TSH: Thyroid-stimulating hormone
fT4: Free thyroxine
anti-TPO: Anti thyroid peroxidase antibodies
TR abs: Thyroid receptor antibodies
FDH: Familial dysalbuminemic hyperthyroxinemia
RTH: Resistance to thyroid hormone
TRβ: Thyroid hormone receptor beta.
